# Systematic review of reported association studies of monogenic genes and bladder cancer risk and confirmation analysis in a large population cohort

**DOI:** 10.1002/bco2.206

**Published:** 2022-12-07

**Authors:** Abrar Mian, Jun Wei, Zhuqing Shi, Andrew S. Rifkin, S. Lilly Zheng, Alexander P. Glaser, James T. Kearns, Brian T. Helfand, Jianfeng Xu

**Affiliations:** ^1^ Program for Personalized Cancer Care NorthShore University HealthSystem Evanston Illinois USA; ^2^ Midwestern University Chicago College of Osteopathic Medicine Downers Grove Illinois; ^3^ Department of Surgery NorthShore University HealthSystem Evanston Illinois USA; ^4^ Department of Surgery University of Chicago Pritzker School of Medicine Chicago Illinois USA

**Keywords:** bladder cancer, germline, monogenic gene, pathogenic mutations, risk

## Abstract

**Objectives:**

To evaluate which of previously reported monogenic genes are associated with increased bladder cancer risk, we reviewed published papers on associations of genes and bladder cancer risk and performed a confirmation study of these genes in a large population‐based cohort.

**Subjects and methods:**

A systematic review of published papers prior to June 2022 was performed first to identify all genes where germline mutations were associated with bladder cancer risk. The associations of these candidate genes with bladder cancer risk were then tested among 1695 bladder cancer cases and 186 271 controls in the UK Biobank (UKB). The robust SKAT‐O, a gene‐based analysis that properly controls for type I error rates due to unbalanced case–control ratio, was used for association tests adjusting for age at recruitment, gender, smoking status, and genetic background.

**Results:**

The systematic review identified nine genes that were significantly associated with bladder cancer risk in at least one study (*p* < 0.05), including *MUTYH*, *MSH2*, *MSH6*, *MLH1*, *ATM*, *BRCA2*, *ERCC5*, *TGFB1* and *CHEK2*. When pathogenic/likely pathogenic mutations were aggregated within each gene, the association was confirmed for three genes in the UKB at *p* < 0.0056 (Bonferroni correction for nine tests), including *CHEK2*, *ATM* and *BRCA2*, all also known to be associated with hereditary breast cancer. Suggestive evidence of association was found for two other genes, including *MLH1* (*p* = 0.006) and *MSH2* (*p* = 0.007), both known to be associated with Lynch syndrome. Among these five genes, the bladder cancer risks range from 1.60 (*ATM*) to 4.88 (*MLH1*), and mutation carrier rates in cases range from 0.06% (*MSH2*) to 2.01% (*CHEK2*).

**Conclusion:**

This study provides statistical evidence for association of previously reported genes and bladder cancer risk and has clinical utility for risk assessment and genetic counselling.

## 1 INTRODUCTION

Urothelial carcinoma of the bladder (bladder cancer) poses a substantial burden in the United States, with over 83 000 cases and 17 000 deaths occurring in the year 2021 alone.^1^ Although environmental factors such as tobacco smoking and exposure to certain chemicals have been associated with bladder cancer risk,^2^ inherited factors also contribute to its susceptibility. Observational studies suggested that individuals with a positive family history of bladder cancer are twice as likely to develop the cancer than those without.^3^ Heritability for bladder cancer was estimated at 30% from twin studies.^4^ More specifically, genome‐wide association studies (GWAS) in the last decade identified 14 independent bladder cancer risk‐associated SNPs.^5^ In addition, several monogenic genes, including those involved in Lynch syndrome, were also reported to be associated with bladder cancer risk.^6^, ^7^, ^8^, ^9^, ^10^, ^11^, ^12^, ^13^ However, compared to breast, colorectal and prostate cancer where many monogenic genes have been conclusively implicated,^14^, ^15^, ^16^ statistical evidence for association of monogenic genes with bladder cancer risk is less well‐established and inconclusive.

Multiple factors may contribute to the current status of association between monogenic genes and bladder cancer. Statistical power to detect the association is limited for bladder cancer due to its relatively low prevalence. The challenge is further compounded by the low frequency of pathogenic mutations in monogenic genes. Their frequency, even aggregated for various mutations, is typically <1% in cases and controls. Although the availability of large population‐based biobanks presents an opportunity to overcome the challenges of small sample size, common statistical methods such as the Fisher's exact test are inadequate. These methods have inflated type I error rate for unbalanced case–control ratios (e.g., the ratio is typically smaller than 1:100 for bladder cancer) and cannot adjust covariates such as age, gender, smoking status and genetic background.^17^ These challenges, however, can be overcome by a newly developed gene‐based method, called robust SKAT‐O.

The primary objective of this study is to assess the validity of reported monogenic genes for their association with urothelial carcinoma of the bladder (bladder cancer). We first systematically reviewed the published studies on association of monogenic genes and bladder cancer risk and identified candidate genes where at least one positive association was reported. We then performed a confirmation study for these genes in a large biobank using the robust SKAT‐O.

## 2 SUBJECTS AND METHODS

### 2.1 Systematic review

We searched PubMed for papers published between 1993 and June 2022 using the keywords of ‘urothelial carcinoma’, ‘bladder cancer’, ‘germline’ and ‘mutations’. The title and abstract of identified papers were reviewed. Three reviewers (A.M, B. H and J.X), working collaboratively, were involved in the review. The inclusion criteria of studies are (1) case–control design, (2) sample size of >100 bladder cancer cases and (3) statistical test for association between germline mutations and bladder cancer. The exclusion criteria are (1) editorial, letter, commentary, review, meta‐analysis, (2) case report, (3) somatic mutations, (4) GWAS/polygenic risk score, (5) not relevant to the current study (i.e., studies on animal model, functional analysis, therapeutic targets, etc.) or (6) studies limited to upper tract urothelial carcinoma (UTUC). Those three reviewers also worked collaboratively on the data collection, reviewing the entire text, tables, figures and supplementary data for study design, sample size and association results for the remaining papers. All included articles were reviewed for certainty assessment by additional team members. Genes that were significantly associated with bladder cancer risk (*p* < 0.05) in at least one paper were subjected for a confirmation study.

### 2.2 Confirmation study

Study subjects were from the UK Biobank (UKB) with available whole‐exome sequencing (WES) data. The UKB, initiated in 2014, is a large prospective cohort of individuals from across the United Kingdom aged 40 to 69 at recruitment (Application Number: 50295).^18^ Extensive phenotypic and genomic information is available for each participant in the UKB, including disease diagnosis, questionnaire, biological measurements, lifestyle indicators and biomarkers in blood and urine. Bladder cancer diagnosis was based on records of inpatient diagnosis, national cancer registry and self‐report. Patients with International Statistical Classification 9 (ICD9) codes of 1880, 1882, 1884, 1886, 1888, 1889 or 2337 and ICD10 codes of C67.0, C67.1, C67.2, C67.3, C67.4, C67.5, C67.6, C67.7, C67.8, C67.9 or D090 were classified as bladder cancer cases. At the time of this analysis, approximately 40% UKB subjects had WES data, including 1695 bladder cancer cases and 186 271 controls (Table 1). Information for cystectomy and radiotherapy delivery is obtained based on Office of Population Censuses and Surveys (OPCS) four codes M34 and X65. Bladder cancer‐specific death was obtained from death registries. Stage and grade of bladder cancer is not available in UKB; therefore, cases of aggressive bladder cancer were defined as those who underwent cystectomy or died of bladder cancer.

**TABLE 1:** Characteristics of study subjects in the UKB^a^

			Association test	
Variables	Cases^b^	Controls	Test	*p*‐Value
No. (%) subjects	1769 (0.88%)	198 776 (99.12%)	N/A	N/A
Age at recruitment, median (IQR) and yr.	63 (59–67)	58 (50, 63)	*t* = 29.02	8.88E‐185
Male gender, no. (%)	1305 (73.77%)	88 778 (44.66%)	χ^2^=599.27, df = 1	<2.2e‐16
Races, no. (%)			χ^2^=17.22 df = 2	1.82E‐04
White	1698 (95.99%)	186 429 (93.79%)		
Black	11 (0.62%)	3210 (1.61%)		
Other racial groups	60 (3.39%)	9137 (4.60%)		
Age at diagnosis, median (IQR) and yr.	66 (58.9–71.6)		N/A	N/A
Cystectomy, no. (%)	59 (3.33%)		N/A	N/A
Died of bladder cancer, no. (%)	182 (10.29%)		N/A	N/A

^a^ Only subjects with WES data are included.

^b^ Case is defined by ICD9 (1880, 1882, 1884, 1886, 1888, 1889, 2337), ICD10 (C67.0, C67.1, C67.2, C67.3, C67.4, C67.5, C67.6, C67.7, C67.8, C67.9, D090) of inpatient diagnosis, cancer registry and self‐report (1035).

Germline variants of studied genes were from the WES data provided by the UKB (PLINK format). All nonsynonymous changes (missense and nonsense) in the targeted genes were extracted. Pathogenic/likely pathogenic (P/LP) mutations were annotated following the general sequence variant interpretation (SVI) guidelines of the American College of Medical Genetics (ACMG).^19^ Mutations classified as P/LP were considered as ‘pathogenic’ in this study.

The primary association test was a gene‐based analysis for pathogenic mutations using the robust SKAT‐O.^17^ This method adjusts single‐variant score statistics through the use of saddle point approximation (SPA) and efficient resampling (ER) and then aggregates the adjusted statistics. The SPA and ER help to precisely calculate the reference distribution of the single‐variant score statistics, thereby properly controlling for type I error rates, even in the scenario where case–control ratio is 1:99. The association test was performed adjusting for several covariates, including age at recruitment, gender, smoking status (ever smoking vs. never smoking) and genetic background (top four principal components provided by the UKB). A *p* < 0.0056 was considered significant based on the Bonferroni correction for nine independent tests (0.05/9).

Crude odds ratio (OR) and 95% confidence interval (CI) were also estimated for each gene using the Fisher's exact test.

## 3 RESULTS

### 3.1 Systematic review

**FIGURE 1:**
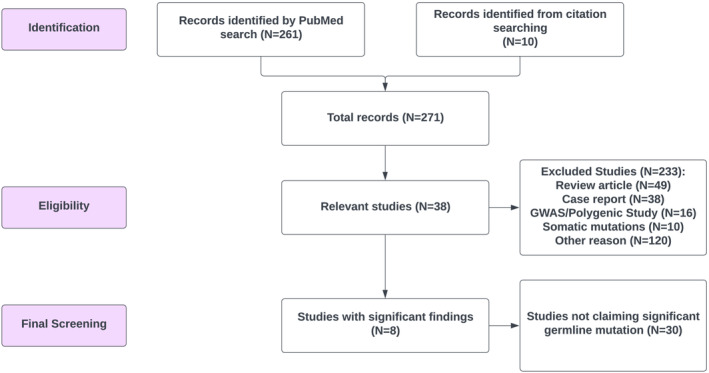
Flow chart for systematic review of published papers on association of germline mutations of monogenic genes and bladder cancer risk.

A PubMed search in June 2022 identified 271 papers on the associations of germline mutations with bladder cancer risk (Figure 1). After excluding ineligible papers related to this study, 38 papers remained for detailed review. Based on these papers, nine genes were reported to be significantly associated with bladder cancer risk (*p* < 0.05) in at least one study, including *MUTYH*, *MSH2*, *MSH6*, *MLH1*, *ATM*, *BRCA2*, *CHEK2*, *ERCC5* and *TGFB1*. The sample size, odds ratio (OR), *p*‐value and reference are listed in Table 2. Most of these genes are known to be associated with hereditary cancer syndromes such as Lynch syndrome (*MSH2*, *MSH6*, *MLH1* and *PMS2*), hereditary breast and ovarian cancer (HBOC) syndrome (*BRCA2*) and hereditary breast cancer (*ATM* and *CHEK2*).

**TABLE 2:** Reported genes associated with bladder cancer risk

Gene	Studies	No. case/control	OR (95% CI)	*p*‐Value	Additional studies with null finding
*ATM*	Nassar et al.	12/827	3.8 (1.8–8.3)	0.02	Zeng et al.
*BRCA2*	Nassar et al.	16/867	5.7 (3.2–9.6)	2.80E‐06	Zeng et al., Perlowska et al.
	Carlo et al.	9/80	3.7 (1.5–7.8)	<0.003	
*CHEK2*	Zlowocka et al.	44/416	1.9 (1.3–2.7)	0.0003	Zeng et al., Naslund‐Koch et al.
	Nassar et al.	11/862	0.2 (0.1–0.4)	5.70E‐07	
*ERCC5*	Na et al.	2/98	n/a	0.01	
*MLH1*	Nassar et al.	10/957	15.9 (4.4–67.7)	7.30E‐05	Zeng et al.
*MSH2*	Nassar et al.	33/969	15.5 (7.1–32.7)	4.40E‐15	Zeng et al.
	Carlo et al.	8/80	4.6 (1.8–9.8)	<0.001	
*MUTYH* ^a^	Vogt et al.	4/276	7.2 (2–18.4)	<0.05	Nassar et al.
	Win et al.	9/5158	19 (3.7–97)	n/a	
*MSH6*	Zeng et al.	269/214020	5.63 (2.75–11.49)	4.94E‐05	Nassar et al.
*TGFB1*	Chen et al.	33/65	2.45 (1.89–3.16)	0.004	Zeng et al.

^a^ Biallelic carriers.

### 3.2 Confirmation study

A gene‐based association test of these nine genes with bladder cancer risk was tested in the UKB using the robust SKAT‐O (Table 3). Based on aggregated P/LP mutations (Supporting information, Table S1), three genes reached study‐wise significance level (*p* < 0.0056), including *CHEK2* (*p* = 8.75E‐05), *ATM* (*p* = 4.95E‐04) and *BRCA2* (*P* = 0.005). In addition, suggestive evidence of the association was found for two other genes at a nominal *p* < 0.05, including *MLH1* (*p* = 0.006) and *MSH2* (*p* = 0.007). No significant statistical evidence was found for the remaining four genes, including *MSH6* (*p* = 0.08), *MUTYH* (*p* = 0.80), *ERCC5* (*p* = 0.90) and *TGFB1* (*p* = 0.84).

**TABLE 3:** Gene‐based analysis of reported genes in the UKB (Whites)

	Bladder cancer (1685 cases, 185 672 controls)				
	P/LP mutations		No. (%) P/LP mutation carriers		
	*p* ^a^	No. mutations	Cases	Controls	OR (95% CI)
*CHEK2*	6.29E‐05	52	34 (2.02%)	1886 (1.02%)	2.01 (1.42–2.83)
*ATM*	5.32E‐04	226	11 (0.65%)	754 (0.41%)	1.61 (0.89–2.93)
*BRCA2*	0.006	195	13 (0.77%)	700 (0.38%)	2.05 (1.18–3.56)
*MLH1*	0.005	32	4 (0.24%)	91 (0.05%)	4.85 (1.78–13.22)
*MSH2*	0.005	26	1 (0.06%)	38 (0.02%)	2.90 (0.40–21.14)
*MSH6*	0.09	90	3 (0.18%)	237 (0.13%)	1.40 (0.45–4.36)
*TGFB1*	0.79	9	0 (0.0%)	19 (0.01%)	0
*ERCC5*	0.88	1	0 (0.0%)	2 (0.0%)	0
*MUTYH*	0.85	59	32 (1.9%)	3557 (1.92%)	0.99 (0.70–1.41)
*MUTYH* ^b^			s1 (0.06)	29 (0.02)	3.80(0.52–27.91)

^a^ Robust SKAT‐O adjusting for age, gender, smoking status, and genetic background (top four principal components).

^b^ >1 allele.

Mutation carrier rates in these five genes were rare in both cases and controls. The gene with the highest mutation carrier rate was *CHEK2* (2.01% in cases and 1.02% in controls). In contrast, *MSH2* had the lowest mutation carrier rate (0.06% in cases and 0.02% in controls). Pathogenic mutations in these five genes were associated with moderate bladder cancer risk, with the crude OR ranging from 1.60 (95% CI: 0.88–2.91) for *ATM* to 4.88 (95% CI: 1.76–13.04) for *MLH1*.

At the individual P/LP level, although none reached the study‐wise significant after adjusting for 790 multiple tests (*p* = 6.33E‐05), 30 pathogenic mutations in these nine genes had *p* < 0.05 (Supporting information, Table **1** and Table **2**). The mutation with the strongest *p*‐value was V2424G of *ATM* (*p* = 1.10E‐04). The mutation carrier rate was 0.24% in cases and 0.02% in controls; crude OR was 14.4 (95% CI: 4.84, 42.87). Three recurrent mutations in cases were found for *CHEK2*: W93X (*p* = 6.11E‐04), c.1462‐2A > G (*p* = 1.09E‐03), and E64K (*p* = 0.01).

Association of these genes with age at diagnosis (Supporting information, Table S3) or aggressiveness of bladder cancer (defined as those who underwent cystectomy or died of bladder cancer) was also assessed (Supporting information, Table S4). Pathogenic mutations in *MLH1* were significantly associated with early age diagnosis (51.85 vs. 64.34, *p* = 0.01). The age at diagnosis for the single *MSH2* mutation carrier was 47.5. Pathogenic mutation rate of *ATM* was significantly higher among aggressive bladder cancer patients (7/208 = 3.37%) than nonaggressive bladder cancer patients (4/1487 = 0.27%), OR = 12.91 (95% CI: 3.71–44.50), *p* = 8.15E‐05. A trend of higher carrier rate of *ATM* V2424G in aggressive bladder cancer patients (2/208 = 0.96%) than nonaggressive bladder cancer patients (2/1487 = 0.13%) was observed, *p* = 0.08.

## 4 DISCUSSION

By systematically reviewing published papers on the association of germline genes with bladder cancer risk and validating reported candidate genes in a large population‐based cohort, we confirmed the association for three genes at the study‐wise significance level (*p* < 0.0056), including *CHEK2*, *ATM* and *BRCA2*. All the three genes are known to be involved in hereditary breast cancer risk.^10^ In addition, we found suggestive evidence of association for two genes at a nominal *p* < 0.05, including *MLH1* and *MSH2*. Both of these genes are known to cause Lynch syndrome.^10^ These key findings provide needed statistical evidence for previously reported bladder cancer‐associated genes and offer guidance for bladder cancer risk assessment in the general population and clinic. For example, genetic counselling for mutations carriers of these genes on cancer risk may extend to bladder cancer and consider screening tests for bladder cancer, including identifying haematuria with a urine dipstick or microscopic urinalysis, urine cytology and tests for urine biomarkers.

Several important and novel aspects of our study approach and results are noted. First, this is a population‐based study and includes unselected bladder cancer patients. Therefore, results from this study not only can be generalized to all bladder cancer patients in the population (hereditary and sporadic, as well as aggressive and nonaggressive) but, more importantly, reduces selection bias in many previous studies where patients were recruited from hospitals, cancer family registry and genetic testing companies.^6^, ^7^, ^8^, ^9^, ^10^, ^11^, ^12^, ^13^ For example, in studies where patients were recruited from those undergoing genetic testing due to family history of hereditary cancer syndromes such as Lynch syndrome and HBOC,^6^ higher mutation carrier rates of cancer syndrome genes are expected in all relatives compared to that of the general population. The higher carrier rates may simply reflect the genetic relationship among family members, rather than implicate their roles in bladder cancer susceptibility. Similarly, results from hospital‐based studies may be more relevant to aggressive subsets of bladder cancer patients.

Second, the same sequencing method and mutation annotation pipeline were used for both cases and controls in our study. This approach considerably reduces false positives due to potential heterogeneity (methods and genetic background) between cases and controls. For example, several published studies compared mutation carrier rate of bladder cancer cases recruited for their studies with that of external public database such as Exome Aggregation Consortium (ExAC).^6^, ^8^, ^11^ Potential differences in sequencing methods (depth, quality controls and annotation pipelines) and genetic background (even within the same ancestry group) between the cases and population controls may contribute to the different mutation carrier rates.

Third, we adopted a novel gene‐based method (robust SKAT‐O) that can overcome several major challenges of commonly used gene‐based methods, including inflated type I error rate for unbalanced case–control ratios and inability to adjust covariates such as age, gender, smoking status and genetic background.^17^ Based on a simulation study, the type I error rates in unbalanced case–control ratios (1:49 and 1:99) were considerably higher in other gene‐based methods.^17^ However, with the case–control ratio of extremely high in our study (1:110), inflated type I error rate remained a concern, although at a modest level.

Lastly, our results not only are consistent with previous findings but also provide additional insights in bladder cancer susceptibility genes. Published studies to date have suggested urothelial carcinoma is a major extracolonic malignancy component of Lynch syndrome caused by pathogenic variants in mismatch repair genes.^20^ Specifically, higher penetrance of Lynch syndrome genes for UTUC (renal pelvis/calyceal cancer and ureteral cancers) was consistently found. The penetrance for urothelial carcinoma of the bladder from sparse data, however, is much lower and controversial. Hence, results from this population‐based study design, together with robust SKAT‐O analysis, provide evidence to the literature and suggest bladder cancer is an extracolonic malignancy component of Lynch syndrome. On the other hand, our finding of stronger statistical evidence for genes known to be associated with hereditary breast cancer (*CHEK2*, *ATM* and *BRCA2*) is novel. It added critical data to the debate on association between bladder cancer risk and genes in DNA damage repair and signalling pathways beyond mismatch repair pathway.^21^

Additional factors may also contribute to the different results between the current and previous studies. In particular, relatively small sample size of bladder cancer patients in studies, including our study, limits statistical power to detect true associations (i.e., false negatives). The rarity of pathogenic mutations in genes further limits the statistical power and can lead to inconsistent findings among studies. For example, in the largest study to date, evaluating 23 hereditary cancer genes among 214 020 subjects from three cohorts (including UKB),^10^ Zeng and colleagues reported that pathogenic mutation in *MSH6* was significantly associated with bladder cancer risk. The meta‐analysis' OR was 5.63 (2.75–11.49), *p* = 1.33E‐05. However, the association was noticeably different among three large sub cohorts, with OR of 8.30 (2.33–29.54), 18.98 (4.32–83.30) and 2.28 (0.79–6.61) in the Electronic Medical Records and Genomics Sequencing (eMERGEseq), Hereditary Cancer Registry (HCR) and UKB, respectively. Additional studies with larger number of bladder cancer cases are needed to obtain more consistent results.

The importance of establishing gene–disease associations is emphasized by ClinGen, a National Institutes of Health (NIH)‐funded resource dedicated to building a central resource that defines the clinical relevance of genes and variants for use in precision medicine and research.^22^ ClinGen recommends an evidence‐based framework for evaluating the clinical validity of gene–disease associations, including evidence from case–control studies. Although the framework is developed for Mendelian diseases, this concept is also applicable to complex diseases with incomplete penetrance such as cancer. In breast cancer, two large case–control studies assessed the association for 34 commonly tested breast cancer genes and found statistical evidence for only eight genes.^23^, ^24^ Similarly, in prostate cancer, we tested association for 10 genes recommended by the National Comprehensive Cancer Network (NCCN) guidelines for germline testing and found statistical evidence for four genes in the UKB.^25^ Established gene–disease associations for breast and prostate cancer have been shown to be instrumental for germline testing of these two types of cancer in clinic. To date, however, few similar studies have been reported for bladder cancer. In the American Urological Association/Society of Urologic Oncology joint guidelines of 2020, no germline testing is currently recommended.^26^ Results from this population‐based study provide an important piece of evidence for establishing gene–disease associations.

The finding for the association between *ATM* V2424G and bladder cancer risk is worth noting. This recurrent missense change is relatively common in cases (0.24%) and confers a strong risk for bladder cancer (crude OR = 14.4 (95% CI: 4.84, 42.87) from a case–control analysis), especially aggressive bladder cancer (crude OR = 12.91, 95% CI: 3.71–44.50) from a case–case analysis. It is predicted as a deleterious mutation by multiple proteins in silico tools and is nonfunctional in multiple different protein assays.^27^, ^28^ Furthermore, the Hereditary Breast, Ovarian and Pancreatic (HBOP) Variant Curation Expert Panel classified it as a pathogenic mutation based on the ACMG criteria.^29^ Although confirmation of this mutation is needed in large studies, this finding further substantiates *ATM*'s role in bladder cancer risk. Furthermore, implication of a specific variant makes it simple for risk assessment and genetic counselling in clinic.

Our study, however, has several limitations. First, only 40% of UKB subjects with available WES data were analysed in the current study. The WES data from the remaining 60% UKB were recently released, and we are in the process of analysing the data. Second, because the variant annotation was based on WES data, potential mutations in promoters and untranslated regions (UTRs) are likely missed. Furthermore, we were unable to evaluate large‐sized germline deletions and insertions in this study. Third, due to the limited bladder cancer‐related clinical and histological information available in the UKB, our study focused primarily on bladder risk based on case–control data. Association of germline mutations with bladder cancer progression or drug treatment response should be evaluated using large bladder cancer cohorts with detailed clinicopathological, drug treatment and follow‐up data. Fourth, with the primary goal of confirming previously reported genes in the literature, we did not perform a comprehensive analysis of all genes in the genome. Although this effort is feasible with the available WES data, larger sample sizes are still needed to obtain statistical power (require more stringent study‐wise significance due to multiple tests). Fifth, functional analysis for identified P/LP mutations was performed. Although this is beyond the scope of this study, it is a major limitation. Finally, it is noted that approximately 95% of study subjects in this study are White; mutations that are unique to minority populations may be overlooked. Large studies conducted within minority populations including Black are needed.

In conclusion, we provided statistical evidence for association of previously reported genes and bladder cancer risk in a large population‐based cohort. The implicated genes may help urologists for risk assessment and genetic counselling of bladder cancer.

## DISCLOSURE OF INTERESTS

None.

## AUTHOR CONTRIBUTIONS

Xu take responsibility for the integrity of the data and the accuracy of the data analysis. **Concept and design**: Xu and Helfand. **Data analysis**: Mian, Wei and Shi. **Manuscript draft**: Mian and Xu. **Critical revision of the manuscript for important intellectual content**: Mian, Wei, Shi, Rifkin, Zheng, Glasser, Kearns, Helfand and Xu. **Supervision**: Xu.

## Supporting information


**Table S1.** P/LP mutations in the nine reported bladder cancer‐risk‐associated genes
**Table S2.** Variant‐based analysis for reported genes in the UKB (Whites)
**Table S3.** Association of mutation carrier status and age at bladder cancer diagnosis in the UKB (Whites)
**Table S4.** Pathogenic mutations of 9 candidate genes in the UKB bladder cancer cases (Whites)
